# Arsenic in Herbal Kelp Supplements: Concentration, Regulations, and Labeling

**DOI:** 10.1289/ehp.10360

**Published:** 2007-12

**Authors:** Daniel Fabricant

**Affiliations:** Scientific and Regulatory Affairs, Natural Products Association, Washington, DC, E-mail: dfabricant@naturalproductsassoc.org

“Case Report: Potential Arsenic Toxicosis Secondary to Herbal Kelp Supplement” by Amster et al. (2007) is fundamentally flawed, both scientifically and with regard to the regulation of dietary supplements.

Amster et al. (2007) claimed to have found “detectable levels of arsenic in eight of the nine kelp herbal supplements, ranging from 1.59 ppm to 65.5 ppm by dry weight (1.59, 2.28, 9.55, 9.97, 10.5, 24.1, 34.8, and 65.5 ppm),” with a median of 10.23 ppm. In this instance, concentrations are irrelevant without disclosing the mass of the capsules. This would allow for calculation of the potential exposure to arsenic; any valid scientific argument on toxicity has to be based on exposure levels and daily intake, not on concentration.

For example, if we applied a 50-mg mass as the capsule mass, this would equate to arsenic concentrations of 0.0795, 0.114, 0.478, 0.499, 0.525, 1.21, 1.74, and 3.275 μg/capsule, respectively. With a serving of one capsule per day, this is well below the normal daily intake cited by Amster et al. (2007):

Nonoccupationally exposed individuals [had] an average total (inorganic and methylated) arsenic intake of 40 μg/day. U.S. dietary intake of inorganic arsenic has been estimated to range from 1 to 20 μg/day (Schoof et al. 1999).

A 500-mg capsule, in effect multiplying the daily intake 10-fold, would still result in all the products below the average daily total intake of 40 μg as cited by Amster et al. (2007).

The glaring omission of the mass of the capsules and the subsequent presentation of the data as a concentration allowed Amster et al. (2007) to provide a provocative story and headline. Once the real-world metrics are applied, however, the fog is dispersed and these numbers are obviously well within the numbers the authors cited as daily intake values. These data are no longer provocative and make it impossible for kelp supplements to be painted as “unsafe” as the authors suggested.

Amster et al. (2007) also were not diligent in researching kelp supplements, and they overlooked key references. One important oversight is the European Pharmacopoeia (2006), which contains a monograph on kelp supplements, with guidelines on arsenic concentration in kelp. The European Pharmacopoeia sets a limit of 90 ppm total arsenic in kelp. Pharmacopoeial monographs are developed over the course of years by experts in the field. The sheer fact that this reference was not cited by Amster et al. (2007) further reveals their ignorance on the subject.

The application of the Food and Drug Administration (FDA) tolerance level for arsenic as residue in muscle meat of chicken and turkey, and in eggs (FDA 2006) in terms of concentration used by Amster et al. (2007) is also not applicable just on product mass alone. For instance, a 4-oz serving of turkey is converted to 113398.0924 mg. Therefore, a 2-ppm limit is applicable and logical based on the mass of the product. This serving of 4 oz turkey at a 2-ppm concentration would obviously result in exponentially greater exposure to arsenic (approximately 2,300 times greater) than a 50-mg kelp dietary supplement capsule at a 2-ppm concentration. To imply that there is toxicity associated with anything—be it a food, pharmaceutical, or dietary supplement—without applying the appropriate metrics is irresponsible and potentially damaging, as well as confusing, to the consumer who may benefit from that product.

In addition to the inappropriate use of metrics, Amster et al. (2007) did not differentiate between the different species of arsenic present in the kelp samples. This differentiation is significant since as the authors themselves present, “In most cases the toxic moiety is presumably trivalent arsenic in the form of inorganic arsenious acid (arsenite).” In fact, the California Clean Drinking Water Act of 1986 (California Environmental Protection Agency 1996), commonly referred to as “Proposition 65,” sets limits only on inorganic arsenic compounds (oxides). This limit is set at 10 mg/day. The California Proposition 65 limit was determined by taking the no observed effect level, which is defined as “the highest level at which a chemical can be administered to an organism without any adverse effect (for example upon health, growth, development, reproductive capacity or lifetime) being observed” (California Environmental Protection Agency 1996), and then dividing by 1,000. In addition, the Food Chemicals Codex (1996) has set a limit of 3 ppm inorganic arsenic. There is no limit for total or organic arsenic compounds. The absence of blood arsenic at the time of poisoning from the study (Amster et al. 2007) is also a relevant and questionable deficiency.

With respect to supplement regulation, supplements must be accurately labeled as mandated by the Dietary Supplement Health and Education Act of 1994 (DSHEA 1994) and actively monitored by the FDA. Regulatory action is taken when and where appropriate. The FDA, on a number of occasions, has stated that the DSHEA provides all the legislative authority needed to regulate dietary supplements. In testimony before the House of Representatives Committee on Government Reform, Robert E. Brackett (Center for Food Safety and Applied Nutrition, FDA) stated that the

FDA regulates the safety, manufacturing, and labeling of dietary supplements, while [the Federal Trade Commission] has primary responsibility for regulating the advertising of these products.” (FDA 2005)

In conclusion, contrary to the viewpoint of Amster et al. (2007), dietary supplements are in fact regulated, have a well-established history of safety, and are essential to the health of the nation.
